# Biology of *Anthonomus testaceosquamosus* Linell, 1897 (Coleoptera: Curculionidae): A New Pest of Tropical Hibiscus

**DOI:** 10.3390/insects13010013

**Published:** 2021-12-22

**Authors:** Alexandra M. Revynthi, Yisell Velazquez Hernandez, Maria A. Canon, A. Daniel Greene, German Vargas, Paul E. Kendra, Catharine M. Mannion

**Affiliations:** 1 Department of Entomology and Nematology, University of Florida, Homestead, FL 33031, USA; yvelazquez@ufl.edu (Y.V.H.); malejandra1@ufl.edu (M.A.C.); anthonygreene@ufl.edu (A.D.G.); german.vargas@ufl.edu (G.V.); cmannion@ufl.edu (C.M.M.); 2 USDA-ARS, Subtropical Horticulture Research Station, Miami, FL 33158, USA; paul.kendra@ars.usda.gov

**Keywords:** invasive pest, hibiscus bud weevil, artificial diet, China rose hibiscus, IPM, life history

## Abstract

**Simple Summary:**

Although native to northeastern Mexico and southern Texas, the hibiscus bud weevil (HBW), *Anthonomus testaceosquamosus* Linell 1897, was recently discovered infesting hibiscus in south Florida in 2017. During outbreak events, HBW feeding on hibiscus buds has been found to significantly affect the marketability of the crop. Therefore, it is vital that an integrated pest management (IPM) program be developed for this pest in order to mitigate the economic loss to the hibiscus industry of south Florida. However, a comprehensive understanding of the HBW’s biology is critical to the development of such a program. In this study, we sought to determine how temperature and diet affect the life history of the HBW. Four temperatures were tested 10, 15, 27 and 34 °C. Life cycle completion was found to only occur at 27 °C, but weevils developed equally as fast on hibiscus buds as on an artificial diet. Adult HBW could survive solely on pollen, but reproduction did not occur. Without water at 27 °C, HBW survived for ≈15 days; survival times reached nearly 30 days when water was accessible. Our results suggest that if left unmanaged, the HBW may cause significant economic damage to the hibiscus industry. We provide a foundation for future research endeavors that aim to better manage this weevil in south Florida.

**Abstract:**

Originating in northeastern Mexico and southern Texas, the hibiscus bud weevil (HBW), *Anthonomus testaceosquamosus* Linell 1897, was discovered infesting China rose hibiscus (*Hibiscus rosa-sinensis* L.) in south Florida in May 2017. Although the biologies of the congeneric boll weevil, *A. grandis* Boheman 1843, and pepper weevil, *A. eugenii* Cano 1894 are well documented, no data are available regarding the biology of HBW. Here, we present a comprehensive study on the biology of this pest when reared at 10, 15, 27 and 34 °C and on different food sources. This weevil has three larval instars and its life cycle was completed only at 27 ± 1 °C. Weevil development was similar on an artificial diet when compared with a diet of hibiscus buds. Adult HBW could survive solely on pollen, but reproduction did not occur. Without water, HBW survived for ≈15 days; survival times reached nearly 30 days when water was accessible. Our results suggest that if left unmanaged, HBW has the potential to cause significant economic damage to the hibiscus industry. Given that a comprehensive understanding of a pest’s biology is critical for development of effective integrated pest management, our results provide a foundation for future research endeavors to mitigate the impact of this weevil in south Florida.

## 1. Introduction

The hibiscus bud weevil (HBW) (*Anthonomus testaceosquamosus*, Coleoptera: Curculionidae) is a small (≈4 mm) insect that infests China rose hibiscus (*Hibiscus rosa-sinensis* L., Malvales: Malvaceae). It originates in northeastern Mexico and southern Texas [1] and has been associated with multiple hosts within the family Malvaceae [1,2]. Female weevils oviposit their eggs inside hibiscus flower buds, inserted close to the anthers. Upon emergence, larvae feed on pollen and remain in the flower bud until they reach adulthood [3]. In Texas, heavy infestations on different varieties of tropical hibiscus resulted in bud drop, thereby decreasing the marketability of the plants [3]. In May 2017, HBW was detected infesting hibiscus in south Florida for the first time [4]; by the spring shipping period of 2019, HBW outbreaks were already responsible for large economic losses to the state’s hibiscus industry.

The discovery of HBW in south Florida is of particular concern due to the importance of the hibiscus industry in the area. Florida is the number one hibiscus producing state, of which most is grown in south Florida (including Miami-Dade County). Approximately 20% to 25% of plants sold from Miami-Dade County are hibiscus, and this ornamental is shipped throughout the North American continent. As of 2017, the market value of ornamental plants in the county was 697 million (farmgate price) [5]. Therefore, the Florida Department of Agriculture and Consumer Services, Division of Plant Industry (FDACS-DPI), is now regulating this pest to curtail its spread. Currently, if HBW is detected at a nursery, the grower must sign a compliance agreement requiring that all plants be weevil-free prior to shipping. Hibiscus growers have a narrow shipping window of 3 months in the spring of each year, from March through June. Any losses incurred during this critical period can be devastating to these growers, and to the Florida industry as a whole.

Despite frequent insecticide applications and the implementation of sanitation practices (i.e., collection and destruction of fallen buds), hibiscus growers remain unable to control HBW populations. Therefore, it is vital that an integrated pest management (IPM) program be developed for this pest to mitigate economic losses to the hibiscus industry of south Florida. However, a comprehensive understanding of a pest’s biology is critical for the development of such a program. Although close relatives of HBW such as the cotton boll weevil, *A. grandis*, and the pepper weevil, *A. eugenii*, have been studied extensively [6,7,8,9,10], little information is available for the HBW aside from an initial FDACS-DPI Pest Alert [4] and a University of Florida Fact Sheet [11]. Consequently, we investigated important biological parameters regarding the HBW life cycle. Specifically, we assessed the effects of temperature and diet on HBW development and fecundity. Here, we present for the first time a comprehensive study on the biology of this pest under different feeding regimes and rearing temperatures.

## 2. Materials and Methods

### 2.1. Weevil Colony

#### 2.1.1. On Hibiscus Buds

Hibiscus buds infested with weevil larvae were received from a local FDACS-DPI inspector after being collected from a nursery in Homestead, Florida, in April 2019. Infested buds were placed in mesh cages (30.5 × 30.5 × 30.5 cm, BioQuip Products Inc., Compton, CA, USA) with fresh hibiscus buds (*H. rosa-sinensis* var. Painted Lady). Upon adult emergence in cages, fresh buds were added twice per week, and old buds were removed and maintained in plastic containers (20.5 × 19 × 37 cm Rubbermaid, Atlanta, GA, USA) until adult emergence. The lid of each container had a hole (5 cm diameter) covered with fine mesh (100 μm diameter) for ventilation. Adult weevils that emerged from the plastic containers were subsequently transferred to a new mesh cage containing fresh hibiscus buds. The colony was maintained in incubators (Percival I-36LL, Percival Geneva Scientific, Williams Bay, WI, USA) at 27 ± 1 °C, 12:12 h L:D and 60% RH. 

#### 2.1.2. On Artificial Diet

A generalist diet used for mass rearing of the pink bollworm, *Pectinophora gossypiella* Saunders (Lepidoptera: Gelechiidae) [12,13] was used to explore an alternative rearing method of the HBW. Diet was placed and compressed into 24-well cell culture plates (Falcon^®^, Fisher Scientific, Pittsburgh, PA, USA), and 70 eggs were individually placed in each well. Eggs were obtained by offering adult females hibiscus buds and allowing them to oviposit for 24 h. A small piece of Parafilm^®^ (Bemis Company, Inc., Neenah, WI, USA) added between the base and the lid allowed for sufficient ventilation without diet desiccation. To simulate conditions within a hibiscus bud, plates were kept in the dark at 27 ± 1 °C and 60% RH until adult emergence. Eggs were monitored daily, and larval feeding was confirmed visually through a change in body coloration (light pink). Emerged adults (F_1_ developed on diet) were placed in a large plant culture dish (100 × 40 mm, SPL Life Sciences, Pro Lab Supply Corp., Hialeah, FL, USA) sealed with a lid containing one large (5 cm diameter) hole covered with fine mesh (100 μm diameter) and one small hole (2 mm diameter) plugged with cotton wool to provide moisture and maintain humidity levels. All adults were provided with fresh hibiscus buds, pollen, and diet twice per week and were kept at 27 ± 1 °C, 12:12 h L:D and 60% RH. 

### 2.2. Instar Determination and Immature Development on Hibiscus Buds and Artificial Diet

To determine the number of instars in the HBW life cycle, an egg cohort was prepared. The cohort was created by allowing the F_1_ adults that had developed on the diet (see Section 2.1) to feed and oviposit on hibiscus buds. After 24 h, the buds were replaced, and the old buds were opened to extract the oviposited eggs. The eggs were individually placed in cell culture plates containing diet and monitored daily. All plates were kept in the dark at 27 ± 1 °C and 60% RH. Hatched eggs were scored and monitored for the entire duration of the larval stage. Each day, a group of 10 larvae was collected and individually introduced to an Eppendorf tube (1.5 mL) with 0.1 mL of 70% ethanol. In total, 10 groups of 10 larvae were collected—one group for each developmental day. Measurements of the head capsule width and length were taken for each larva using a Keyence Microscope (Nikon^®^ SMZ1270, Nikon Instruments Inc., Melville, NY, USA).

To estimate the egg-to-adult development of HBW at different temperatures on its natural host, hibiscus buds were used. Twenty pairs of HBW were released in a cage containing 40 non-infested hibiscus buds and were allowed to feed and oviposit for 24 h. The buds were then removed from the cage and inspected for the presence of eggs. Twenty eggs were randomly selected and individually inserted (via scalpel) onto the anthers of a new, non-infested bud. The buds were then placed individually in Petri dishes (Corning™ 100 × 15 mm, Fisher Scientific, Pittsburgh, PA, USA). The lid of each Petri dish contained two larger holes (21 mm diameter) covered with fine mesh (100 μm diameter) and one smaller hole (2 mm diameter) plugged with cotton wool to provide moisture and maintain humidity levels. The Petri dishes were kept in incubators at 10, 15, 27 or 34 ± 1 °C, 12:12 h L:D and 60% RH. Buds were inspected daily, and each day the developmental stage was recorded. The experiment was terminated once all individuals reached adulthood or died. Tested temperatures were selected to cover the temperature fluctuations that typically occur in south Florida throughout the year. From November through April (dry season) the average minimum temperature is 15 °C, while the average maximum temperature is 27 °C. From May through October (rainy season) the average minimum temperature is 27 °C, while the average maximum temperature is 34 °C (Climatestotravel.com). Photoperiod was set at 12:12 h L:D because throughout the year Florida has on average a 12 h day length.

To evaluate the egg-to-adult development of HBW on the artificial diet, nine egg cohorts (*n* = 129 eggs) were used. Eggs were individually placed in cell culture plates containing diet and monitored daily. Since this experiment was designed to assess the artificial diet as an alternative food source for rearing of HBW, weevil development was only evaluated at 27 ± 1 °C, 60% RH under dark conditions. 

### 2.3. Reproduction and Longevity on Hibiscus Buds and Artificial Diet

Given that temperatures of 10, 15 and 34 °C prohibited HBW life cycle completion, reproduction and longevity experiments were only conducted at 27 ± 1 °C, 12:12 h L:D and 60% RH. To determine the mating system of HBW, 14 virgin females and 14 virgin males were isolated and individually placed in a Petri dish with a hibiscus bud. To ensure that weevils were virgin, they were isolated at the pupal stage. Every 24 h, the number of eggs laid in the bud was destructively scored and a new hibiscus bud was added. Eight days later, seven females were paired with seven males. The remaining 14 individuals were kept separately as control groups. Daily oviposition was scored until all weevils died. 

To estimate population growth and adult longevity, 20 pairs (24 h-old individuals) of HBW were randomly selected and offered a hibiscus bud as feeding and oviposition substrate. Daily oviposition was scored for each female, and adults were observed until death. Additionally, egg viability was calculated by rearing all oviposited eggs from 10 females for a week. Adult longevity was assessed by individually placing ten virgin female and male (24 h-old) weevils in a Petri dish containing a hibiscus bud as a food source. The weevils were monitored daily until they died, and the buds were replaced three times per week. Longevity was calculated for both males and females.

Emerged adult females (F_2_) that developed on the artificial diet from the previous experiment were paired with emerged adult males from the same generation. Twenty pairs were randomly selected to receive either a hibiscus bud or the artificial diet as feeding and oviposition substrate. Weevils feeding and reproducing on hibiscus buds were kept in small plastic Petri dishes (GSC Go Science 50 × 15 mm, Fisher Scientific, Pittsburgh, PA, USA) containing a hole (10 mm diameter) covered with fine mesh (100 μm diameter). Petri dishes (Falcon^®^ 1006, 50 × 9 mm, Fisher Scientific, Pittsburgh, PA, USA) containing weevils in the artificial diet (compressed and covering the bottom of the dish) treatment group featured 10 holes (1 mm diameter) in the lid to allow for ventilation. All adult pairs were kept at 27 ± 1 °C, 12:12 h L:D and 60% RH. Daily oviposition was scored for each female, and adults were observed until death. Longevity was calculated for both males and females. To estimate the sex ratio of the progeny (F_3_), six egg cohorts were created by individually isolating each egg that was oviposited within hibiscus buds in cell culture plates containing diet. Eggs were reared to adulthood, the gender of each individual was scored, and fertility was also calculated. All plates were kept in the dark at 27 ± 1 °C and 60% RH.

### 2.4. Life Table and Population Parameters

Life tables and population parameters were calculated using oviposition and longevity data from females that: developed and reproduced on hibiscus buds, developed and reproduced on an artificial diet, and developed on an artificial diet and reproduced on hibiscus buds at 27 °C [14]. Life table parameters included the pivotal age (x) for the age class in days, the number of surviving females (l_x_) at the age class x, the number of living females born per female in each age interval (m_x_) and the total number of female births in each age interval (l_x_m_x_). These parameters were used to calculate the net reproductive rate (R_o_), the cohort generation time (T), the intrinsic rate of increase (r_m_), the doubling time (DT), and the finite rate of increase (λ) [14,15].

### 2.5. Adult Survival on Pollen and without Food Source 

An experiment was conducted to assess the effect of a pollen-only diet on adult weevil reproduction and survival. Twelve pairs (24 h-old individuals) of adult weevils were randomly selected and given a hibiscus (var. Painted Lady) stamen (≈3 cm in length) in which the pollen had not yet been released (i.e., the anthers were closed). Adult weevils were monitored daily, and stamens were replaced three times per week. Adult longevity and daily oviposition were scored until all individuals died.

To estimate how long adult weevils can survive without food and with or without water, 20 adult weevils (24 h-old males and females) were randomly selected and individually placed in Petri dishes as described in Section 2.1. Half of the weevils (10 individuals) were provided with wet cotton wool, while the other half were not provided with access to water. Adult longevity was scored until all individuals died.

### 2.6. Data and Statistical Analyses

#### 2.6.1. Instar Determination

To estimate the number of instars that occur during HBW development, measurements of head capsule widths were subjected to mode testing using the excess mass test as described in Ameijeiras-Alonso [16]. Dyar’s constant was calculated to define head capsule growth [17]. Mean head capsule widths among different instars were assessed using a generalized linear model (GLM) with quasi-Poisson distribution, and contrasts (Tukey adjustment) were assessed with the estimated marginal means method using the “emmeans” package in R [18]. 

#### 2.6.2. Development on Hibiscus or Artificial Diet 

The effect of food source (hibiscus buds or artificial diet) and temperature on larval development (in days) was assessed using linear regression models (LMs). Individual models included the number of days required for development for either egg, 1st–3rd instar larvae, pupae, and eggs-to-adults as the response variable, and food source and temperature as explanatory variables. Contrasts among explanatory variables were assessed through general linear hypothesis testing (glht of the lsmeans package with the ‘Tukey’ adjustment of *p* values; [19]). Since weevils only reached the pupal and adult stages at 27 °C, food source was the lone explanatory variable in pupal and egg-to-adult development models.

#### 2.6.3. Reproduction and Longevity on Hibiscus or Artificial Diet 

For models with count data as the response variable (fecundity, oviposition, longevity), preliminary, separate generalized linear models (GLMs) were constructed for each dataset using Poisson, quasi-Poisson, and negative binomial error distributions to account for overdispersion. Selection of the final model was based on the error distribution that provided the best fit for each dataset. Hereafter, only the information pertaining to the final model for each dataset will be discussed. Negative binomial GLMs were used to assess the effect of food source on HBW development and reproduction. Individual models included either fecundity (eggs per female per day), fertility (proportion of egg hatch), pre-oviposition, oviposition and post-oviposition period as the response variable and food source (buds, diet or diet + buds) during development and reproduction as the lone explanatory variable. Weevil sex ratio was tested using a Chi-square test (α = 0.05) while the effect of isolation status (paired or solitary), gender, and their interaction (explanatory variables) on adult longevity (in days; response variable) was estimated using a GLM with a negative binomial distribution. The effect of gender, food source (hibiscus buds or artificial diet), and their interaction (explanatory variables) on adult weevil longevity (in days; response variable) was also estimated using a GLM with a quasi-Poisson distribution. Contrasts among explanatory variables were assessed with the estimated marginal means method with a Tukey adjustment of the probabilities [18].

#### 2.6.4. Life Table and Population Parameters 

The effect of food source (buds, diet or diet + buds; explanatory variable) during development and reproduction on the reproductive parameters (R_o,_ T, r_m_, DT, and λ; response variables) was assessed using LMs. Contrasts among food sources were assessed through general linear hypothesis testing.

#### 2.6.5. Survival on Pollen and without Food Source 

Survival on pollen was estimated using a GLM with a quasi-Poisson distribution. Weevil longevity (in days) was the response variable and gender was the explanatory variable. Contrasts among explanatory variables were assessed with the estimated marginal means method with a Tukey adjustment of the probabilities [18]. Survival with or without access to water was assessed using a GLM with a quasi-Poisson distribution. Weevil longevity (in days) was the response variable and water (with or without), gender and their interaction were the explanatory variables. Contrasts among explanatory variables were assessed with the estimated marginal means method with a Tukey adjustment of the probabilities [18]. All statistical analyses were performed using R version 4.1.1 [20].

## 3. Results

### 3.1. Instar Determination and Development on Hibiscus Buds and Artificial Diet

**Figure 1:**
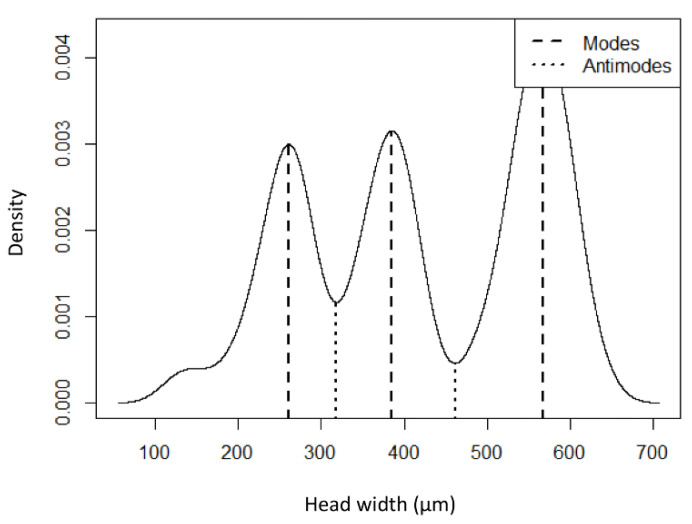
Density distribution of larval head capsule widths of the hibiscus bud weevil (*Anthonomus testaceosquamosus*). The graph presents the three unimodal peaks as estimated by the excess mass test as described in Ameijeiras-Alonso [16]. Each peak represents a larval instar.

**Figure 2:**
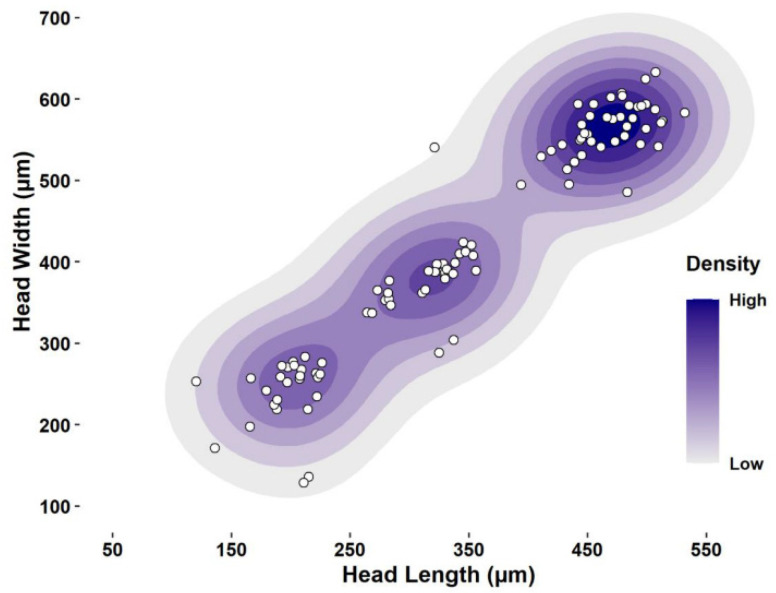
Correlation of larval head capsule widths and lengths of the hibiscus bud weevil (*Anthonomus testaceosquamosus*). The three different groups correspond to the three larval instars.

Measurements of head capsule widths from 98 larvae that developed on artificial diet at 27 °C, had three unimodal peaks (Figure 1) and were strongly and positively correlated with head capsule lengths (Figure 2). Excess mass test for mode testing confirmed that HBW has three larval instars (excess mass = 0.04, *p* = 0.47, reject H_a_: true number of modes is greater than 3). Mean head capsule widths differed significantly among the three instars (GLM: χ^2^ = 1156.1, df = 2, *p* << 0.001), and Dyar’s constant was calculated as 1.48 between first and second instars and between second and third instars (Table 1). To verify that no instars were omitted, the logarithm of head capsule widths was plotted against the number of instars, which resulted in a straight line. The regression equation obtained from these data was Ln y = −10.507 + 2.116x and was highly significant (*p* << 0.0001, r^2^ = 0.90, *n* = 96). 

At 10, 15 and 34 °C, the HBW was not able to complete its life cycle (Table 2). Temperature significantly affected the development of the egg (LM: F = 5850.94, df = 3, *p* << 0.001), second (LM: F = 28.97, df = 2, *p* << 0.001) and third instar stages (LM: F = 9.72, df = 2, *p* << 0.001). Eggs failed to hatch at 10 °C while development through the third instar was observed at 34 °C. At 27 °C, eggs hatched within 3.35 ± 0.3 days (±SE) on average while the entire life cycle (egg-adult) was completed in 15.78 ± 0.8 days (±SE) on average (Table 2).

Weevil development on artificial diet at 27 °C (16.47 ± 0.3 days) was similar to hibiscus buds (15.78 ± 0.83 days) (LM: F = 0.65, df = 1, *p* = 0.42). The egg and first instar stages were significantly shorter on artificial diet in comparison to weevils that developed on buds (Egg: LM: F = 5.1, df = 1, *p* = 0.02; First instar: LM: F = 4.79, df = 1, *p* = 0.02). The third instar and pupal stages did not vary significantly between the two food sources (Third instar: LM: F = 0.54, df = 1, *p* = 0.47; Pupa: LM: F = 0.22, df = 1, *p* = 0.64). On the artificial diet, weevils reached adulthood within 16 days (Table 2). 

### 3.2. Reproduction and Longevity on Hibiscus Buds and Artificial Diet

Virgin females did not oviposit, but when females were offered a mate, oviposition started within the first 24 h. Weevils that developed on artificial diet and oviposited on the diet or in buds had significantly lower fecundity than weevils that developed and reproduced on only hibiscus buds (LM: F = 122.85, df = 2, *p* << 0.001). Pre-and post-oviposition periods were significantly longer when weevils developed on the diet than on buds (pre-oviposition: GLM.NB: χ^2^ = 41.55, df = 2, *p* << 0.001; post-oviposition: GLM.NB: χ^2^ = 46.75, df = 2, *p* << 0.001). However, oviposition period was not affected by the food source (GLM.NB: χ^2^ = 1.64, df = 2, *p* = 0.44) (Table 3). Sixty-two percent of eggs hatched when females had a bud as a substrate. No data are available about the fertility of females that oviposited on the diet (Table 3). 

R_o_, r_m_ and λ of weevils that developed on diet and reproduced on diet or buds were significantly lower compared to females that developed and reproduced on only buds (Ro: LM: F = 42,842, df = 2, *p* << 0.001; r_m_: LM: F = 88,323, df = 2, *p* << 0.001; λ: LM: F = 65,165, df = 2, *p* << 0.001) while T and Dt were significantly higher in these weevils (T: LM: F = 71,425, df = 2, *p* << 0.001; Dt: LM: F = 16,139, df = 2, *p* << 0.001) (Table 4). 

There was a significant interaction between the isolation status (paired or solitary) and the gender of the adult weevils (GLM.NB: χ^2^ = 25.5, df = 1, *p* << 0.001). When weevils were in pairs, males lived longer than females, while isolated females lived longer than isolated males (Table 5A). The sex ratio did not differ significantly from a 1:1 female:male ratio (Chi-square test: χ^2^ = 0.009, df = 1, *p* = 0.93). Females lived significantly longer than males regardless of the oviposition substrate that they were offered (GLM: χ^2^ = 4.6, df = 1, *p* = 0.03) (Table 5B). 

### 3.3. Adult Survival on Pollen and without Food Source

When fed solely on hibiscus pollen, female weevils did not oviposit. Both female and male weevils lived for an average of 30 days (±24 days for females and ±23 days for males) (±SE) (GLM: χ^2^ << 0.001, df = 1, *p* = 0.99). Access to water significantly increased adult longevity (GLM: χ^2^ = 11.16, df = 1, *p* < 0.001). When weevils had access to water, they lived for an average of 28.3 (±3.2) days, while without access to water they lived for an average of 16 (±1.7) days. On average, males lived longer than females, but the difference was not significant (GLM: χ^2^ = 2.46, df = 1, *p* = 0.12). (Table 5C).

## 4. Discussion

The HBW is a newly invasive pest in south Florida for which there is currently only one report that demonstrates its potential impact on the hibiscus industry [3]. Here we present the first comprehensive study on the biology of HBW reared at various temperatures and on various food sources, including its natural host (hibiscus buds), an artificial diet (pink bollworm diet), hibiscus pollen, and only water. Of the temperature regimes evaluated, 27 °C was the most favorable for weevil development. At this temperature, HBW successfully completed its life cycle within 15 days on its natural host (Table 2). These results are consistent with the high weevil populations observed in hibiscus nurseries between March and June. The abundance of flower buds in combination with favorable climatic conditions is conducive for weevil population growth during these months. Since hibiscus plants are shipped nationally and internationally from Miami-Dade County from March through June, the peak numbers of HBW during this critical period pose a serious threat to the Florida hibiscus industry. Growers must ensure that this regulated pest is absent from all hibiscus stock prior to shipment.

In controlled laboratory tests, HBW was not able to complete its life cycle at low (10, 15 °C) or high temperatures (34 °C), suggesting that these environmental conditions are unfavorable for HBW population growth in the field. During the winter months in south Florida, temperatures average 15 °C, but during occasional cold fronts and frost events, temperatures can drop below 0 °C. In this period, hibiscus plants are small, in their vegetative growth stage, and lacking flower buds. During the summer months, however, afternoon temperatures can exceed 34 °C in July and August. During this time, hibiscus cuttings are kept in greenhouses prior to planting. Therefore, environmental conditions in combination with food availability may account for the fluctuations in weevil populations observed in nurseries. It remains unknown whether the HBW has an overwintering form and if yes, which form this is.

Our measurements of HBW head capsule width and length indicated the presence of three larval instars (Table 1), which agrees with other *Anthonomus* species such as *A. grandis* [7,9] and *A. eugenii* [10,21]. Due to the various challenges and the labor required to maintain a laboratory colony with hibiscus buds, we also evaluated an alternative, artificial diet for rearing HBW. We found that although HBW can develop and reproduce on the pink bollworm artificial diet (Table 2, Table 3 and Table 4), its population growth was significantly lower than on hibiscus buds (Table 4). In the congeneric *A. grandis,* the pink bollworm diet was found to be an excellent rearing medium as the wheat germ within the diet stimulated oviposition [22]; this effect was not observed with HBW. Our results are more similar to those reported by Toapanta et al. [10] and Toba et al. [23], whereby *A. eugenii* required more time to develop when reared on an artificial diet than when it was reared on its natural host. Seal and Martin [24] used the artificial cotton boll weevil diet to successfully rear *A.eugenii*. The cotton boll weevil diet and pink boll worm diet are very similar. The main difference is that the former contains cholesterol [22,24]. However, we do not know whether the lack of cholesterol is responsible for the low oviposition of the HBW. Future experiments should test the HBW ability to develop and reproduce on the cotton boll weevil diet. Given these results, we conclude that the artificial pink bollworm diet can serve as an alternative food source for laboratory rearing when hibiscus buds are not available, but hibiscus buds remain the most suitable food source for HBW reproduction.

At 27 °C, population growth and net reproductive rate of HBW (Table 4) were higher than that of *A. grandis* [6,7,9] and *A. eugenii* [10] reared at a similar temperature and photoperiod. Moreover, HBW generation time and doubling time were much shorter in comparison to these other two important agricultural pests [7,10]. These results suggest that if left unmanaged, HBW has the potential to cause significant economic damage to the hibiscus industry. The HBW is widespread in Miami-Dade County in south Florida and has been shown to cause significant injury to hibiscus. The main management practice is chemical control using contact and systemic insecticides. However, whenever feasible, sanitation practices are also being implemented. Collection and destruction of dropped, infested buds have been proven to contribute to the management of weevil populations in nurseries [3].

HBW oviposition did not differ between flower buds and artificial diet and was similar to values reported for *A. grandis* [6,7] and *A. eugenii* [10,24,25]. However, unlike these two and other congeneric weevil species [26,27], HBW oviposits multiple eggs per flower bud. Under laboratory conditions, HBW females were found to oviposit up to 12 eggs per bud, while the maximum number of eggs that has been found per bud in the field is four. Variability in HBW oviposition has also been reported as a function of hibiscus bud size, as Bográn et al. [3] found that buds measuring 0.5–1.5 cm in length were more frequently infested than smaller or larger buds. Although HBW oviposits multiple eggs per bud, only a couple of adults will emerge from each bud. This result may be explained by larval cannibalism. Cannibalism is a common phenomenon across the animal kingdom [28,29], and is well-documented in insects [30] and in the family Curculionidae [31,32]. It can serve as a ‘life boat’ survival mechanism when food is scarce [33], which may be a common occurrence inside small hibiscus buds where the amount of pollen is limited and larvae cannot disperse in search of more pollen. In our experiments it was not possible to observe larval cannibalism because to score daily oviposition we had to destroy the bud and remove all the eggs. Moreover, in weevil development experiments we only inserted one egg per bud or cell. 

In our study virgin HBW females did not oviposit, confirming that this species cannot reproduce through parthenogenesis and mating is required. This result is in accordance with previous research for the congeneric strawberry blossom weevil *A. rubi* (Herbst 1795) [27], *A. eugenii* [10] and *A. grandis* [34], which were found to be incapable of reproduction without mating. Information on reproductive ability can be important in the development of an effective IPM program. Mating disruption has been found to be an important tool for managing lepidopteran pests [35,36], and a similar strategy might be developed for use in HBW management. Additionally, several species within the *Anthonomus* genus have been found to be attracted to a group of commercial lures that consists of male aggregation pheromones and host plant volatiles [37,38,39]. Four components comprise the synthetic male aggregation pheromone, which are known as Grandlures (I–IV). Although the Grandlures were initially designed to attract only *A. grandis* males [36,39,40], both males and females were attracted to these lures in field settings [41]. Pheromone traps broadly used for other *Anthonomus* species have also been used as a pest monitoring tool. In Texas, pheromone traps developed for the cotton boll weevil were evaluated for the detection of HBW adults, but without success [3]. However, the authors stated that this result may be partially due to early season deployment of the traps. Yellow sticky traps have also been used to monitor weevil populations, and were demonstrated to be the most attractive trap for several *Anthonomus* species [42,43,44]. Hence, an important future line of research could focus on developing an HBW-specific lure in combination with an effective trap design and visual cue to assist with monitoring or even mass trapping of this pest in hibiscus nurseries.

Our results concerning adult longevity may be best understood within the context of the evolution of the level of social organization that HBW populations exhibit in natural settings. Under field conditions, weevils display solitary behavior outside of mating events. In this study, isolation status was found to differentially impact adult longevity for males and females (Table 5). Females lived longer when individually isolated than when paired with the opposite sex, whereas for males, the opposite was true. (Table 5A). We hypothesize that male and female fitness would be maximized under different social organization scenarios, with females benefiting from solitary organization and males benefiting from gregarious organization. Our results suggest that sexual conflict in this species may have manifested as sexually antagonistic coevolution, resulting in a system (i.e., solitary organization) that maximizes the fitness of the females over that of the males (i.e., gregarious organization); a likely explanation for the evolution of a solitary level of social organization in the HBW may be attributed to anisogamy [45], although this remains to be experimentally demonstrated. Competing interests between conspecific males and females are common where mating is concerned [46,47], and ultimately arise due to genetic differences between reproductive partners [48]. In yellow dung flies, *Scathophaga stercoraria* L. (Diptera: Scathophagidae), male post-copulatory mate-guarding behavior can reduce sperm competition through the prevention of additional mating events for a female [49,50,51]. While mate-guarding behavior may increase the male’s chances of fertilizing the female’s eggs (i.e., beneficial for the male) [49], sexual conflict arises due to the fact that physical struggles associated with this behavior can injure or even kill the females being guarded [50]. Studies have also shown that mate-guarding behaviors can impede female foraging and predator avoidance abilities [52,53]. Further experiments should be conducted to determine which components of HBW female fitness are compromised when the amount of contact that they have with males is increased. When HBW developed on artificial diets, females lived longer than males regardless of the oviposition substrate offered (Table 5B). In these experiments, weevils were paired, suggesting that the food provided during development can affect adult longevity. It is generally known that food quality can affect organismal life-history investments [54]. In different rearing systems, adult longevity has been found to be affected by the specific components of artificial diets [55,56,57]. Currently, we do not know which component of the pink bollworm diet affected the adult longevity of HBW.

Adult HBW could survive on a diet consisting solely of pollen, but females did not oviposit, which differs from observations on *A. grandis* [8]. HBW could also survive nearly a month on water alone, and approximately half of this time without water (Table 5C). These results indicate that management of this pest poses a significant challenge. Every summer, after the hibiscus shipping period is over and before the new crop is brought in, growers remove hibiscus plants that were not sold and clean the nursery area. Nevertheless, reinfestations occur every year. Our results show that the weevils can survive for a minimum of 2 weeks without water, which may partially explain why we observe these annual reinfestations. Additionally, alternative hosts may contribute to the weevil survival when hibiscus plants are unavailable. Seven wild species/genera within the Malvaceae family have been associated with HBW, and several of these plants are found in Florida [2]. Hence, they might serve as alternative food sources for the weevils. This hypothesis, however, remains to be tested. 

## 5. Conclusions

HBW can successfully complete its life cycle within 2 weeks on *H. rosa-sinensis* at an optimal temperature of 27 °C. Hibiscus buds are a more efficient food source for weevil reproduction than the artificial pink bollworm diet. When buds are not available, weevils are capable of survival on hibiscus pollen and water, but oviposition is sacrificed. The current study generates useful information regarding the biology of a destructive pest of hibiscus, an economically important ornamental cultivated in south Florida. Given that a comprehensive understanding of the biology of a pest is critical for development of an effective IPM program, our results provide a knowledge base for improving management strategies for HBW in Florida nurseries.

## Figures and Tables

**Table 1:** Mean head capsule widths (μm) of larvae of the hibiscus bud weevil (*Anthonomus testaceosquamosus*) at 27 ± 1 °C, 60% RH and 12:12 h (L:D) photoperiod. Within each column, different letters indicate significant differences (Tukey, *p* < 0.05).

Instar	*n*	Width ± SE	Range	Dyar’s Constant
First	29	248.14 ± 8.57a	129–318.37	-
Second	26	383.31 ± 4.65b	318.38–461.38	1.48
Third	43	563.23 ± 5.07c	461.39–633	1.48

**Table 2:** Mean developmental time (days) ± SE of the hibiscus bud weevil (*Anthonomus testaceosquamosus*) under different temperatures and food sources at 60% RH and 12:12 h L:D. Within each column, different letters indicate significant differences (Tukey, *p* < 0.05).

Food Source	Temperature (°C)	Egg (*n*)	First Instar (*n*)	Second Instar (*n*)	Third Instar (*n*)	Pupa (*n*)	Egg to Adult (*n*)
Hibiscus buds	10	78.2 ± 0.55a (20)	-	-	-	-	-
	15	13 ± 1.33b (20)	4.9 ± 0.86a (10)	12.75 ± 2.46a (4)	87 ± 14.01a (3)	-	-
	27	3.35 ± 0.31d (20)	2.6 ± 0.24a (20)	3.73 ± 0.48a (19)	2.05 ± 0.19b (18)	4.1 ± 0.27 (18)	15.78 ± 0.83 (18)
	34	5.5 ± 0.29c (20)	2.53 ± 0.29a (19)	8.92 ± 1.3b (13)	25.5 ± 8.86ac (6)	-	-
Artificial diet	27	2.22 ± 0.05e (129)	1.94 ± 0.05b (128)	3.9 ± 0.08a (128)	4.25 ± 0.23b (128)	4.21 ± 0.07 (128)	16.47 ± 0.3 (128)

**Table 3:** Reproductive parameters for the hibiscus bud weevil (*Anthonomus testaceosquamosus*) when it developed, fed, and reproduced solely on hibiscus buds, on the artificial boll worm diet, or when it developed on the diet and reproduced on hibiscus buds. Within each column, different letters indicate significant differences (Tukey, *p* < 0.05).

Development, Feeding and Oviposition	Fecundity * (*n*)	Fertility ** (*n*)	Pre-Oviposition Period *** (*n*)	Oviposition Period *** (*n*)	Post-Oviposition Period *** (*n*)
Hibiscus buds	5.85 ± 0.48a (20)	55.2 ± 2.32 (10)	4.05 ± 0.4c (20)	40.35 ± 3.53 (20)	4.45 ± 1b (20)
Artificial diet	0.2 ± 0.04b (21)	NA	6.33 ± 0.3b (21)	32.29 ± 3.48 (21)	19.85 ± 3.94a (21)
Artificial diet + Hibiscus buds	0.73 ± 0.57b (20)	62 ± 0.03 (25)	11.35 ± 1.21a (20)	38.45 ± 3.15 (20)	19.85 ± 3.2a (20)

Values are mean ± SE. * (eggs/Female/Day). ** (% hatch). *** Days.

**Table 4:** Life table parameters and 95% confidence intervals for the hibiscus bud weevil (*Anthonomus testaceosquamosus*) when it developed, fed, and reproduced solely on hibiscus buds, on the artificial boll worm diet, or when it developed on the diet and reproduced on hibiscus buds. Within each column different letters indicate significant differences (Tukey, *p* < 0.05).

Development, Feeding and Oviposition	*n*	Net Reproductive Rate (Ro) *	Intrinsic Rate of Increase (rm) **	Generation Time (T) ***	Doubling Time (Dt) ***	Finite Rate of Increase (λ) ***
Hibiscus buds	20	136.73a 135.54–137.91	0.4547a 0.4522–0.4573	10.82a 10.76–10.88	1.52a 1.51–1.53	1.5758a 1.5717–1.5798
Artificial diet	21	7.65c 7.48–7.83	0.0578c 0.0572–0.0584	35.2c 35.03–35.3	11.99c 11.86–12.13	1.0599c 1.0588–1.0601
Artificial diet and Hibiscus buds	20	20.85b 20.46–21.23	0.0841b 0.0834–0.0847	36.09b 35.96–36.09	8.24b 8.18–8.30	1.0877b 1.0871–1.0885

* Female/female. ** Female/Female/Day. *** Day.

**Table 5:** Mean adult longevity (days) of hibiscus bud weevils (*Anthonomus testaceosquamosus*) in different trials addressing their (A) status (paired vs. solitary) when feeding on hibiscus flower buds; (B) development on artificial diet, as they were offered either hibiscus flowers buds or artificial diet as food and oviposition substrate; and (C) access to water when no other food source was offered. Within each trial, columns with different letters indicate significant differences (Tukey, *p* < 0.05).

Trial	Gender	*n*	Longevity ± SE	Max Longevity	Min Longevity
(A) Status					
Paired	Female	20	47.3 ± 4.5a	89	13
	Male	20	111.1 ± 8.4b	169	58
Solitary	Female	10	109.2 ± 12.8b	162	14
	Male	10	86 ± 9.9b	134	42
(B) Food/oviposition source					
Hibiscus buds	Female	20	69.65 ± 6.1a	127	35
	Male	20	61.85 ± 5.9b	115	11
Artificial diet	Female	21	75.62 ± 4.5a	107	30
	Male	21	60.33 ± 5.0b	107	25
(C) Access to water					
With	Female	4	25.5 ± 4.51a	34	14
	Male	6	30.1 ± 4.5b	50	16
Without	Female	6	13.7 ± 1.5a	18	9
	Male	4	19.5 ± 2.9b	28	16

## Data Availability

Data are available at https://doi.org/10.6084/m9.figshare.17323823.v1 (accessed on 3 December 2021).
